# Substrate accumulation and extracellular matrix remodelling promote persistent upper airway disease in mucopolysaccharidosis patients on enzyme replacement therapy

**DOI:** 10.1371/journal.pone.0203216

**Published:** 2018-09-18

**Authors:** Abhijit Ricky Pal, Jean Mercer, Simon A. Jones, Iain A. Bruce, Brian W. Bigger

**Affiliations:** 1 Department of Paediatric Otolaryngology, Royal Manchester Children’s Hospital, Manchester, United Kingdom; 2 Stem Cell and Neurotherapies, Division of Cell Matrix Biology & Regenerative Medicine, Faculty of Biology, Medicine and Health, Manchester Academic Health Science Centre, University of Manchester, Manchester, United Kingdom; 3 Willink Biochemical Genetics Unit, Manchester Centre for Genomic Medicine, St. Mary’s Hospital, Manchester, United Kingdom; 4 Respiratory and Allergy Centre, Institute of Inflammation and Repair, Faculty of Medical and Human Sciences, University of Manchester, Manchester, United Kingdom; Azienda Ospedaliero-Universitaria Santa Maria della Misericordia, ITALY

## Abstract

**Introduction:**

Mucopolysaccharide diseases are a group of lysosomal storage disorders caused by deficiencies of hydrolase enzymes, leading to pathological glycosaminoglycan accumulation. A number of mucopolysaccharidosis (MPS) types are characterised by severe airway disease, the aetiology of which is poorly understood. There is ongoing evidence of significant clinical disease in the long-term despite disease modifying therapeutic strategies, including enzyme-replacement therapy (ERT). To provide a better understanding of this aspect of disease, we have characterised extracellular matrix (ECM) and inflammatory alterations in adenotonsillar tissue samples from 8 MPS patients.

**Methods:**

Adenotonsillar samples from MPS I, IVA and VI ERT treated patients and from a single enzyme naïve MPS IIIA individual were compared to non-affected control samples using quantitative immunohistochemistry, qPCR and biochemical analysis.

**Results:**

Significantly increased lysosomal compartment size and total sulphated glycosaminoglycan (p = 0.0007, 0.02) were identified in patient samples despite ERT. Heparan sulphate glycosaminoglycan was significantly elevated in MPS I and IIIA (p = 0.002), confirming incomplete reversal of disease. Collagen IV and laminin α-5 (p = 0.002, 0.0004) staining demonstrated increased ECM deposition within the reticular and capillary network of MPS samples. No significant change in the expression of the pro-inflammatory cytokines IL-1α, IL-6 or TNF-α was seen compared to control.

**Conclusion:**

This study suggests a role for ECM remodelling contributing to the obstructive phenotype of airway disease in MPS. Current therapeutic strategies with ERT fail to normalise these pathological alterations within adenotonsillar samples. Our findings lend novel insight into the pathological cascade of events, with primarily structural rather than inflammatory changes contributing to the continuing phenotype seen in patients despite current therapeutic regimes.

## Introduction

Mucopolysaccharide diseases are a heterogeneous group of inherited metabolic lysosomal storage disorders with a combined incidence of 1 in 22,000 [1]. They are characterized by deficiencies of specific lysosomal hydrolase enzymes, which result in accumulation of partially degraded glycosaminoglycans (GAGs) and altered cellular function [2]. Pathogenic storage of GAGs, and the subsequent dysfunction from the cellular to systemic level in mucopolysaccharidosis (MPS), manifests as multisystem disease. Patients commonly present with cardiorespiratory, musculoskeletal, visceral and neurocognitive disease [3, 4].

Airway involvement is a well-recognised feature of MPS I, II, IV and VI and significantly contributes to morbidity and premature mortality [5, 6]. The impact of respiratory manifestations on health and wellbeing is highlighted in clinical trials, as measures of airway obstruction and pulmonary function are routinely used as primary or secondary outcomes in interventional trials [7–10]. Current therapeutic regimes aimed at disease modification, including enzyme replacement therapy (ERT) in MPS I Hurler-Scheie (HS) and Scheie, II, IVA and VI and haematopoietic stem cell transplantation (HSCT) in MPS I Hurler (H), have demonstrated organ specific and systemic metabolic correction [11–14]. However, airway disease continues to cause significant complications, with a phenotype that appears to be a combination of structural and inflammatory features [15, 16].

The structural changes observed within the pharynx and laryngotracheal complex present as airway obstruction, most commonly secondary to adenotonsillar hypertrophy and tracheomalacia. A reactive environment has been described on endoscopic examination of the upper airways, with mucosal infiltration within the larynx and trachea and evidence of bronchitis noted within the distal airways [17, 18]. Animal studies have investigated the aetiological mechanisms seen within the musculoskeletal and central nervous system (CNS) identifying inflammation as critical in the degenerative phenotype exhibited [19–21]. Within the CNS of MPS mice, GAGs have been implicated as acting as damage associated molecular patterns (DAMPs) initiating monocyte activation and cytokine cascades [21, 22]. The aetiopathology of airway manifestations is unknown and analysis of tissue specimens from the upper aero-digestive tract of patients with MPS may provide us with novel insight into the disease process.

The airway in MPS provides a potential paradigm of both structural and inflammatory disease within a single organ system. The aetiology of airway deposits and of the inflammatory changes seen within the airway are yet to be elucidated. GAG induced perturbations in mucosal and extracellular matrix (ECM) function or induction of inflammatory cascades are presumed to play a role. However, this has not been demonstrated in MPS. We aim to identify the pathogenic events that continue to cause airway morbidity in the upper airway of MPS by determining and quantifying the structural and inflammatory phenotype of MPS patient tissue in comparison to unaffected controls.

## Methods

### Patient sample collection

MPS patients undergoing adenotonsillectomy for the resolution of symptoms of upper airway obstruction and obstructive sleep apnoea (OSA) at the Royal Manchester Children’s Hospital, UK, were recruited to the study following informed written consent. Tissue samples deemed surplus to clinical requirements were collected from patients undergoing surgery for these clinically indicated procedures under ethical permission 13/NW/0029 (Greater Manchester North Research Ethics Committee) from April 2013 onwards.

Clinical data on sample collection, patient demographics including phenotype, age at commencement of treatment and details of interventions for airway obstruction were documented.

#### Clinical investigations

Overnight sleep oximetry studies were performed in recruited patients during unsedated natural sleep, using sleep oximetry equipment (Pulsox 300i, Konica Minolta) using analysis criteria as previously described [23]. Studies were performed pre-operatively and as per current guidance, to determine the degree of upper airways obstruction [24, 25].

### Analysis of tissue architecture and structure

#### Histology

Haematoxylin and eosin and periodic acid schiff (PAS) histological staining was performed according to standard protocols on identical 5μm sections from patient samples [26].

#### Immunohistochemistry

Patient adenotonsillar samples were mounted in OCT (RA Lamb, Eastbourne, UK) and cut as 5μm cryosections onto glass slides (CM1850, Leica, Wetzlar, Germany). Immunofluorescent staining against human lysosomal associated membrane protein 2 (LAMP2; mouse anti-H4B4 IgG, 5.45μg/ml; developed by August, JT, Developmental Studies Hybridoma Bank, University of Iowa, USA) and heparan sulphate (HS) GAG (mouse anti-F58-10E4 IgM; 14.0μg/ml; Amsbio, UK) were performed using previously described protocols [27, 28]]. Mouse anti-Collagen IV (IgG; 3.32μg/ml; Developmental Studies Hybridoma Bank, University of Iowa, USA) and mouse anti-Laminin-α5 subunit (IgG; 1 in 20; Millipore, UK) were used to demonstrate human ECM proteins as these have previously been identified as the ubiquitous ECM component of adenotonsillar lymphoid tissue [29]. IgG and IgM mouse anti-human and rat anti-mouse antibodies were used as negative controls for each panel. Slides were stained using the Shandon Sequenza staining rack (Fisher Scientific, UK). Briefly frozen sections were fixed in -20°C acetone: methanol (1:1) for 1 minute, washed x 3 with TBS-0.3% Triton X-100, blocked with 10% goat serum, 1% BSA, 0.3% Triton X-100 in TBS for 1 hour at room temperature and incubated with the primary antibody at optimised concentrations overnight in blocking buffer at 4°C. Following a further wash (x4), sections were incubated with the appropriate secondary antibodies, diluted 1:1000 (Alexa 488 or Alexa 594 goat anti-mouse IgG and IgM in human samples, Life technologies, Paisley, UK) followed by 300nM DAPI (Invitrogen) for 15 minutes. Sections were washed, allowed to air dry and mounted with Prolong Gold Antifade mounting medium (Invitrogen) prior to being coverslipped.

#### Microscopy and Image analysis

Images were acquired using the Pannoramic 250 Flash II automated digital scanning microscope (3D Histech Ltd, Budapest, Hungary) using a 20x/0.80 Plan Apo objective for brightfield and fluorescence scanning and a 40x/0.95 Plan Apo objective additionally for fluorescence and viewed using Pannoramic viewer (3D Histech Ltd) [30]. Three sections from each specimen were stained and imaged. From each section, 3 non-overlapping representative fields of view from the superior, central and inferior aspect of each section were chosen based on architecture and a 3x3 grid using the x20 objective from the scanned image of each slide (Pannoramic Viewer, 3D Histech Ltd, Budapest, Hungary). To quantify the degree of staining, images were converted to an 8-bit grayscale TIFF format for analysis using ImageJ software (NIH, USA; http://rsb.info.nih.gov/ij). The mean staining intensity, based on relative grayscale pixel intensity (Arbitary units), from the 3 fields of view from 3 sections per organ in each mouse / individual were calculated [31]. Images from each section were taken at the same exposure setting during the same session.

#### Total tissue GAG assay

Total sulphated GAG quantification in patient samples was performed using the Blyscan Assay (Biocolor Ltd., UK) as previously described [32]. GAG levels were corrected for total protein content and expressed as μg GAG mg^-1^ total protein.

### Determination of inflammation and immune phenotype

#### Real Time PCR

Patient samples of adenotonsillar tissue were placed in RNAlater solution (Sigma, UK) and stored at −20°C at the time of surgery. RNA was isolated for measurement by RT-PCR of IL-1α, IL-6, and TNF-α mRNA expression using TriZOL (Life Technologies, UK) and RNeasy Mini kit (QAIGEN, West Sussex, UK) and treated with Turbo DNase enzyme (Ambion Ltd., UK) to minimise DNA contamination. Quantification was performed using a Nanodrop spectrophotometer and 100 ng of total RNA reverse transcribed into cDNA (Superscript III Reverse Transcriptase, Invitrogen). Taqman gene expression assays (Applied Biosystems, UK) containing sequence specific primers for human IL-1α (Assay ID:Hs00174092_m1), IL6 (Assay ID: Hs0098539_m1) and TNF-α (Assay ID: HS01113624_g1) were used to achieve target specific amplification. GAPDH was used as the endogenous comparator gene. RT-PCR was performed in triplicate on a StepOne Plus Real Time PCR system (Applied Biosystems, UK). Age matched non-affected patient controls were used as calibrator reference samples. The comparative ΔC_T_ method was used to determine relative mRNA expression [33].

### Statistical analysis

Descriptive statistics were calculated for demographics, sleep oximetry and biochemical data. Correlation coefficients were calculated with Pearson’s r and Spearman’s rho for normally and non-normally distributed data sets respectively. Student’s T-test, Mann-Whitney and one way and two-way ANOVA with post hoc analysis by Tukey’s multiple comparisons test were used to identify differences between the characteristics of subgroups. P-values <0.05 were considered significant. JMP v11.0 software (SAS) was used for this analysis. All raw data is provided as supporting information in S1 to S7 Tables.

## Results

### Study demographics

The patients enrolled in this study were those identified as requiring adenotonsillectomy, adenoidectomy or tonsillectomy alone for clinical symptoms of upper airway obstruction. Twelve patients with a mean age of 5.5 years (range 2–12 years) were recruited to the study, composed of 8 MPS patients (2 MPS I HS, 1 MPS IIIA, 3 MPS IVA and 2 MPS VI). All but the MPS IIIA patient were treated with ERT with a median age at commencement of 3 years. The median duration on ERT was 2.2 years (range 0.7–7.5years). Four individuals, without a diagnosis of metabolic disease, were recruited as controls and age and sex matched where possible. A specific history of tonsillitis was sought prior to enrolment, to exclude the potential confounding effect of infection on assessment of inflammatory profile within tissue. Subject demographic, treatment characteristics and sleep study outcomes are presented in Table 1.

**Table 1:** Patient demographics, treatment and tissue characteristics.

Patient No.	MPS phenotype	Gender	Age @ enrolment	Age @ start of ERT	Surgery	Preop ODI4%	DS:CS Ratio
**1**	I HS	M	6.2	4	Revision adenoidectomy for OSA. Previous adenotonsillectomy, 2008	12.7	0.74
**2**	I HS	F	9.5	2	Adenotonsillectomy for OSA	15.3	0.7
**3**	IIIA	M	3.4	-	Adenoidectomy for nasal obstruction and glue ear	12.5	-
**4**	IVA	F	4.2	3	Tonsillectomy for OSA. Previous adenoidectomy 2011	9.0	-
**5**	IVA	M	12.2	8	Adenoidectomy for OSA. Previous tonsillectomy 2009	45.4	-
**6**	IVA	M	4.3	2	Tonsillectomy for OSA	12.1	-
**7**	VI	F	2.0	1	Adenotonsillectomy for OSA	15.1	2.5 (PRE ERT)
**8**	VI	M	9.7	9	Adenotonsillectomy for OSA. Late diagnosis.	-	1.2
**9**	Control	F	4.3	-	Adenoidectomy for nasal obstruction and glue ear	-	-
**10**	Control	F	2.1	-	Adenotonsillectomy for mild OSA	5.1	-
**11**	Control	M	4.9	-	Adenotonsillectomy for mild OSA	1.3	-
**12**	Control	M	3.4	-	Adenotonsillectomy for mild OSA	12.0	-

ERT, Enzyme replacement therapy; Preop ODI4%. Preoperative sleep study oxygen desaturation index 4%; HS, Hurler-Scheie; OSA, Obstructive sleep apnoea.

ERT is not currently available for MPS III

#### Sleep disordered breathing

All but 2 patients underwent overnight sleep oximetry studies, with the most recent pre-surgical study assessed (median: ODI4% 12.3/hour, range 1.3–45.4; 5.2% time spent below 90% oxygen saturations, range 0–18.8%; median nocturnal oxygen saturation 95.1%, range 91.9–99.0%). Six of the 8 (75%) MPS patients had evidence of severe SDB based on previously published criteria (Standards for Services for Children with Disorders of Sleep physiology, 2009) [34].

#### Immunohistochemistry of patient samples

From the 12 recruited patients, 9 adenoid and 8 tonsil samples were processed and analysed. No significant differences were seen in staining intensity between adenoid and tonsillar samples from the same individual or in the group as a whole (p = 0.65). As a result adenotonsillar samples were pooled for analysis and grouped by MPS type, rather than tissue type.

### ECM architecture in MPS patient samples

**Fig 1:**
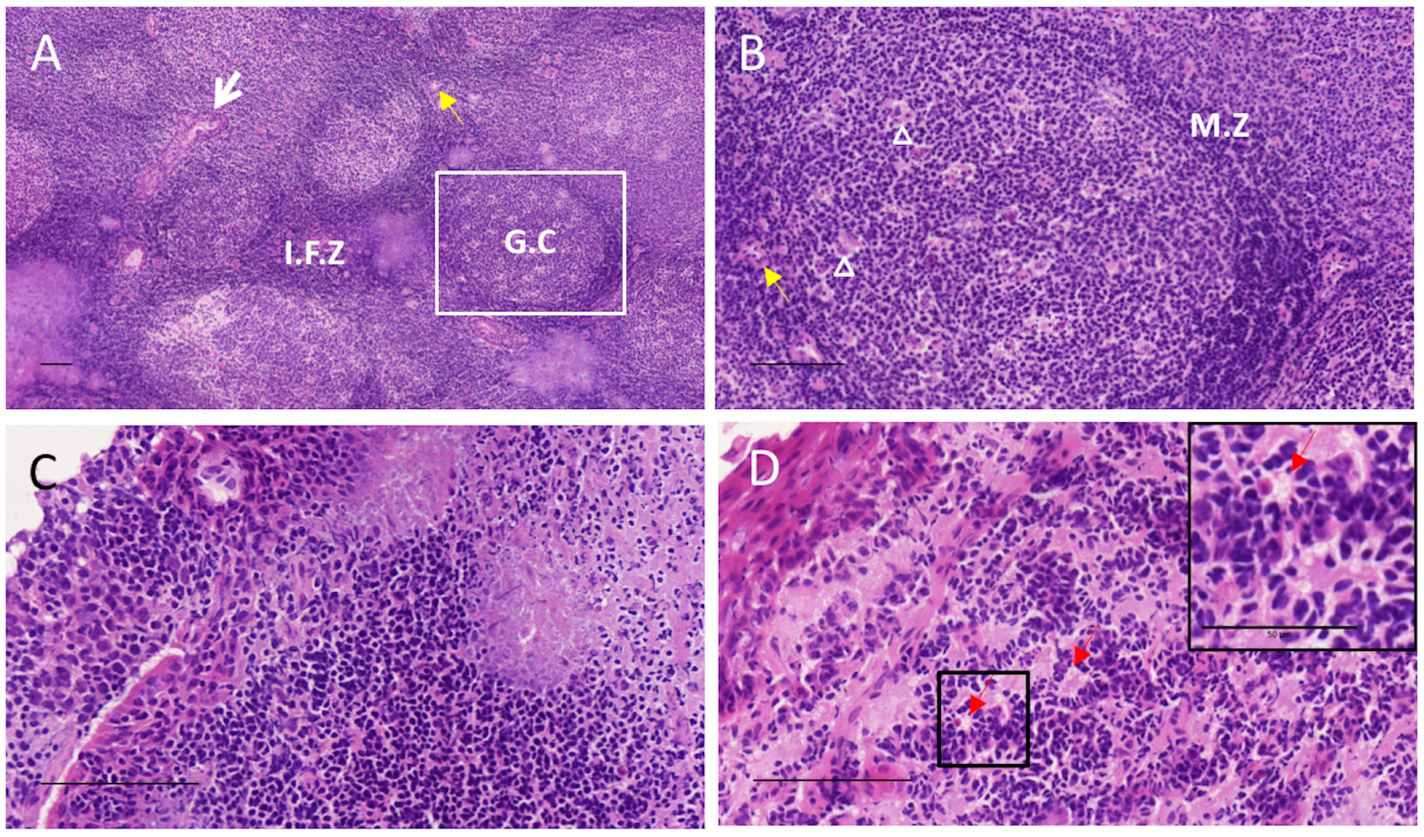
Tissue architecture of tonsillar tissue in control and MPS I patient. Haematoxylin and eosin staining demonstrates lymphoid architecture with (A) low and (B) high power fields of view. G.C, germinal centre, composed of follicular dendritic cells, antigen presenting cells and B-cells; M.Z, mantle zone, composed of a rim of T lymphocytes; I.F.Z, interfollicular zone, containing hyalinsed vascular network of arterioles (yellow arrow); squamous crypt epithelium (white arrow); tingible body macrophages (arrowhead). (C) High power field of view of the subepithelial layer in control patient. (D) Equivalent field of view with magnified inset of subepithelial layer in an MPS I patient, demonstrating histiocytoid cell forms (red arrow). Scale bars represent 100μm except in magnified inset in D (50μm).

Collagen IV and laminin α-5 are known to localise to ECM reticular network and capillary basement membrane (BM). In order to determine architecture and ECM remodelling in adenotonsillar samples, H&E and immunostaining against these proteins was undertaken. The gross microscopic follicular architecture of adenotonsillar lymphoid tissue was preserved in MPS patients. High power magnification identified histiocytoid changes within the cells of the interfollicular zones and subepithelial stroma as shown in Fig 1.

**Fig 2:**
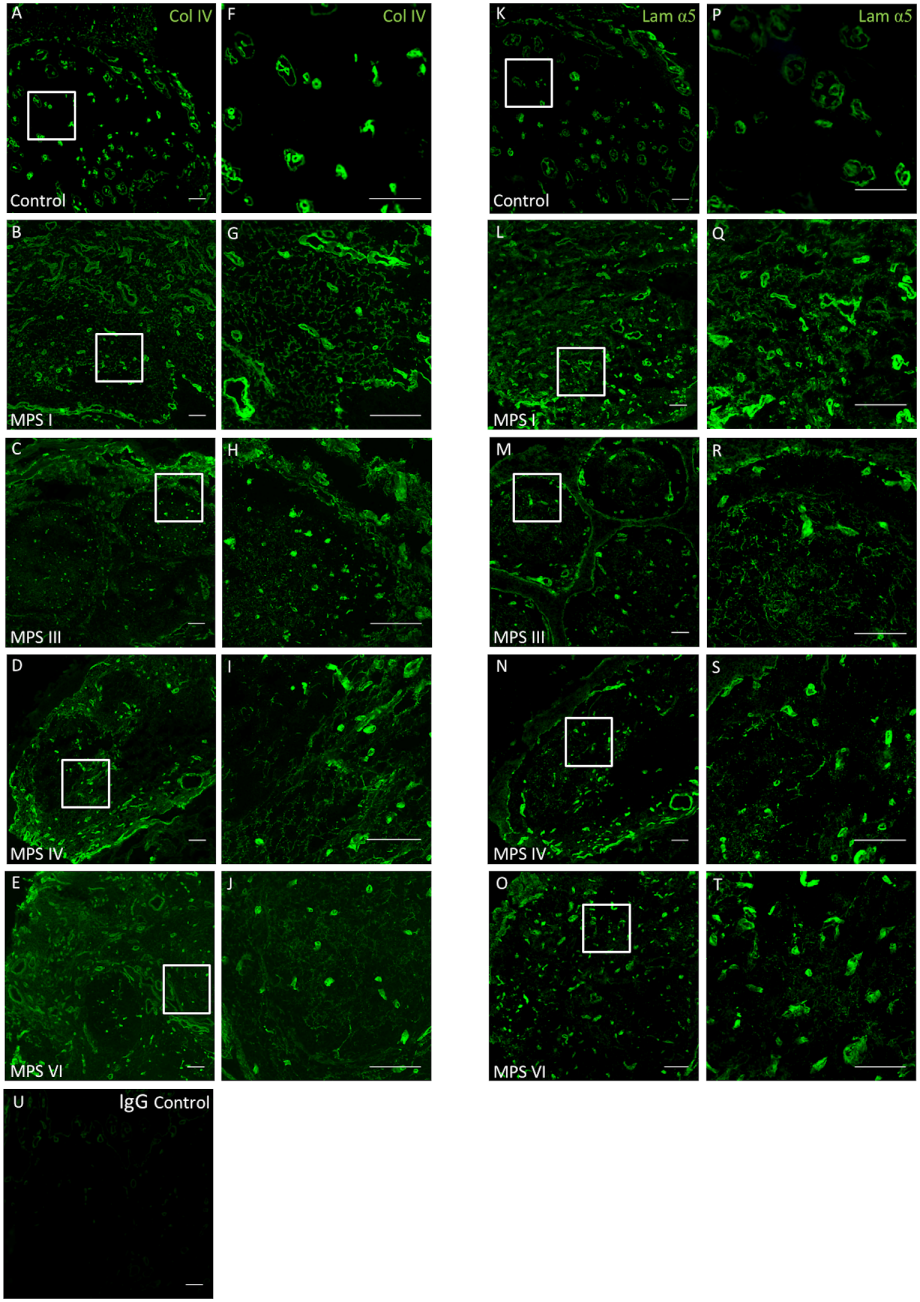
Increased ECM deposition in adenotonsillar tissue of MPS patients. Collagen IV and Laminin α-5 immunoreactivity was quantified in adenotonsillar samples from 8 MPS patients and compared to samples from 4 non-affected control individuals. Scale bar = 100μm. Representative low power (A-E, x20) and high power (F-J, x60) sections demonstrating collagen IV (green) staining in control (A, F), MPS I (B, G), MPS III (C,H), MPS IV (D,I) and MPS VI (E,J) with localisation to the basement membrane (BM) and hyalinised vessel walls. In MPS samples a denser pattern of reticular network staining is demonstrated within the stroma. A similar pattern of staining is demonstrated for laminin α-5 (green). Representative low power (K-O, x20) and high power (P-T, x60) sections in control (K, P), MPS I (L, Q), MPS III (M,R), MPS IV (N,S) and MPS VI (O, T). IgG negative control (U).

Collagen IV and laminin α-5 subunit demonstrated similar staining patterns within all ECM compartments of the adenoid and tonsil (Fig 2). In both the control and MPS samples, the crypt epithelium, hyalinsed arterioles, capillary network and reticular fibre network immunoreacted to the ECM and BM components.

**Fig 3:**
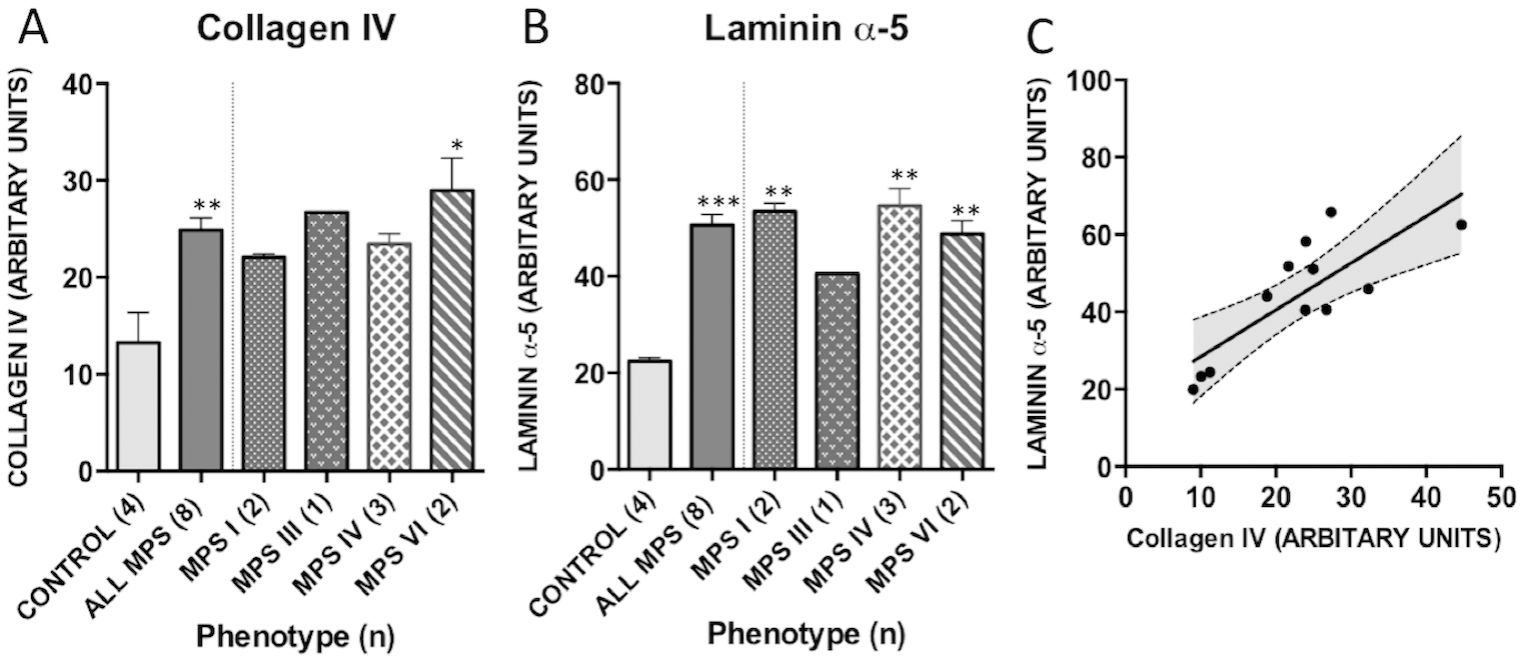
Quantification of collagen and laminin immunoreactivity in patient tissue. 3 fields of view from 3 serial sections were quantified for (A) collagen IV and (B) Laminin α-5 antibody using Image J. Mean grey scale intensity values are presented. Error bars represent SEM and p-values are from one way ANOVA with Tukey’s multiple comparisons test. Significant differences to control are demonstrated with * p<0.05, **p<0.01, ***p<0.001. (C) Collagen IV and Laminin-α5 staining correlate significantly in adenotonsillar tissue samples in individuals (R^2^ = 0.64, p = 0.002).

In samples from the same individual, collagen IV staining correlated strongly with laminin reactivity, suggesting localisation to similar ECM components (R^2^ = 0.64, p = 0.0017, Fig 3). Dual stains were not performed as both antibodies were cross reactive to mouse anti-human IgG serotypes. Quantification of collagen IV and laminin-α5 immunofluoresence from each specimen was compared to control samples (Fig 3). The mean intensity of collagen and laminin staining was increased in adenotonsillar tissue in the MPS cohort as a whole against control samples (collagen IV, p = 0.002; laminin, p = 0.0004). The phenotype effects between individual MPS types compared to non-affected control individuals was noted to be significant in MPS VI for collagen IV (p = 0.02) and MPS I, IV and VI for laminin (MPS I, p = 0.004, IV, p = 0.004, VI, p = 0.008). As only one MPS III sample was available, analysis using a one-way ANOVA was not possible. The increase in staining originates from an increased density of deposition in the reticular fibre meshwork and capillary walls within the stroma of the lymphoid tissue.

#### GAG storage

**Fig 4:**
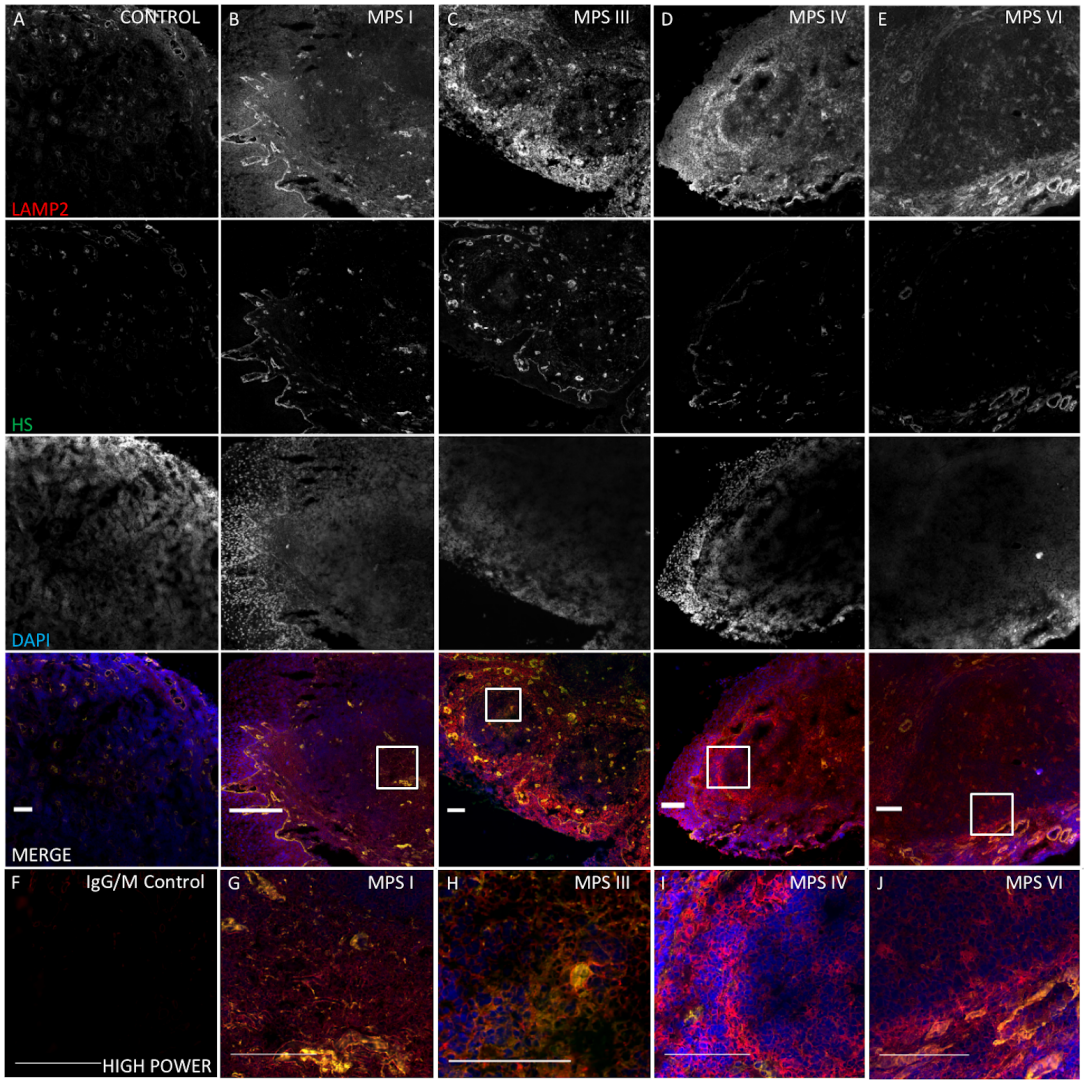
Increased, lysosomal compartment size and HS storage in adenotonsillar tissue of MPS patients. LAMP2 and HS immunoreactivity in representative adenotonsillar samples from 8 MPS patients, compared to samples from 4 non-affected control individuals. Scale bar = 100μm.(A-E) Single overlays and merged images at x 20 in control (A), MPS I (B), MPS III (C), MPS IV (D) and MPS VI (E) demonstrate LAMP2 (Red) is prominent in the IFZ and subepithelial layer and HS localises to basement membrane (BM) and vessel walls. LAMP2 is increased in MPS, while HS is increased in MPS I and III, but not significantly so in the non-HS storing types IV and VI. Nuclei are stained blue with DAPI. (F) IgG and IgM negative control. (G-J) High power fields of view of the germinal centre (x100) in each sample shown in (B-E) demonstrating co-localisation in the reticular network and capillary walls in MPS I and III. HS localised to the arteriole BM in all sample including control.

**Fig 5:**
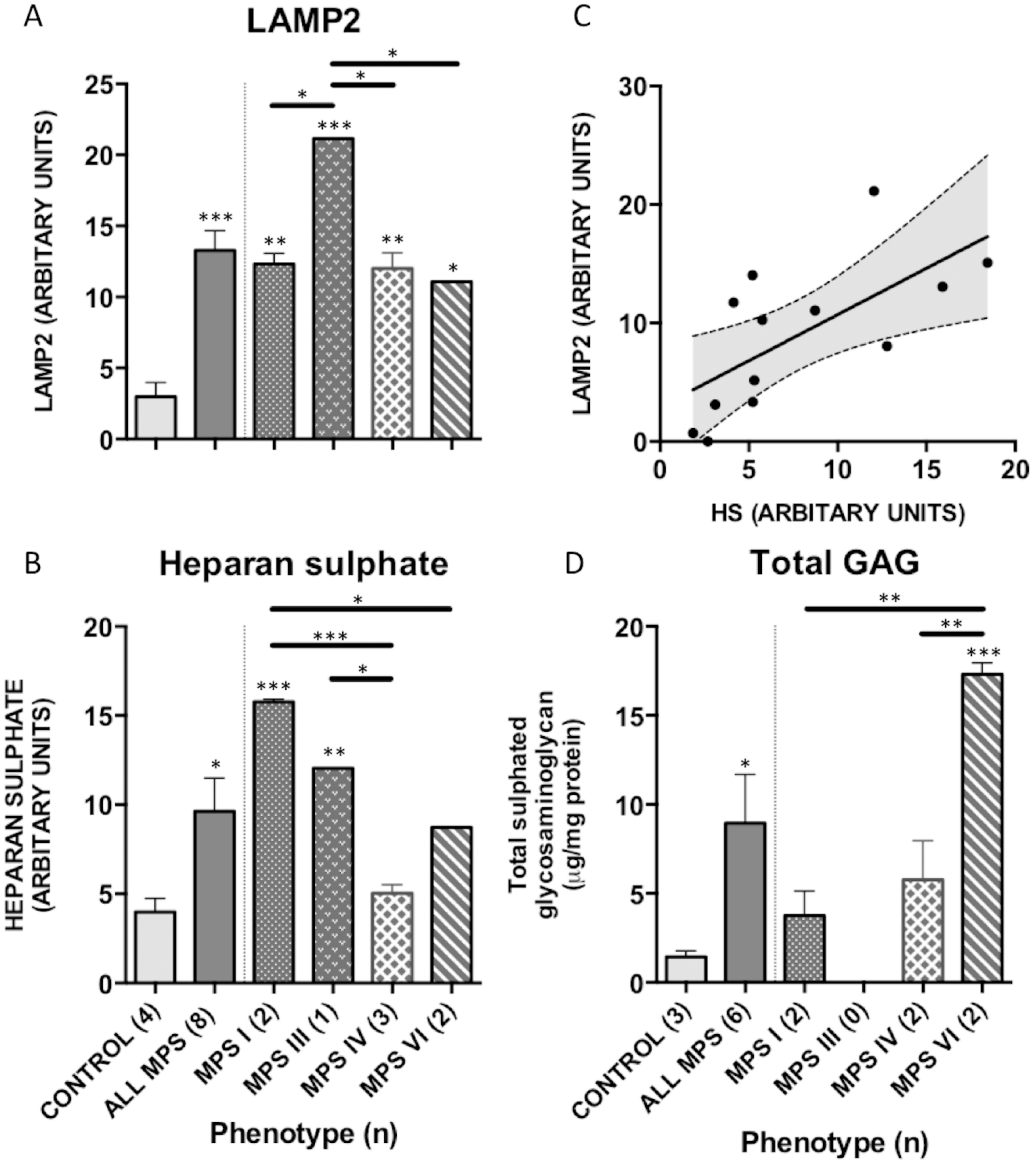
LAMP2 and HS quantification in MPS patient tissue. Three fields of view from 3 serial sections were quantified for (A) LAMP2 and (B) HS using Image J. Mean grey scale intensity values are presented. Error bars represent SEM and p-values are from one-way ANOVA with Tukey’s multiple comparisons test. (C) HS and LAMP2 staining correlate significantly in adenotonsillar tissue in individuals. (D) Blyscan assays were performed in adenotonsillar tissue to quantify total sulphated GAG. GAG is significantly increased in MPS patients compared to control, and in MPS VI compared to MPS I and IV (n for each patient group in parentheses). Significant differences to control are demonstrated with * p<0.05, **p<0.01, ***p<0.001, significant genotype differences between individual MPS types are denoted with a solid bar.

To investigate GAG storage in patient tissue, HS accumulation was assessed with 10E4 antibody immunostaining and total tissue GAG was quantified using the Blyscan assay. HS localised to the basement membrane and vascular endothelial structures of the arteriole network in both control and MPS, in keeping with the structural role of PGs. Co-localisation with LAMP2, to within the lysosomal compartment of reticular matrix and capillary ECM components was more evident in HS storing MPS types I and III (Fig 4). Quantification analysis demonstrated significantly increased staining in adenotonsillar tissue of the MPS cohort over control samples (p = 0.02), with storage in MPS I (p = 0.0002) and MPS III (p = 0.005) responsible for this (Fig 5). As expected, the non-HS storing MPS types IV and VI were not significantly raised over control. In keeping with the above findings, MPS I was noted to have significantly raised HS immunoreactivity over MPS IV (p = 0.0004) and VI (p = 0.02), and MPS III was raised over MPS IV (p = 0.01). Total sulphated GAG, as measured with the Blyscan assay, was raised in all MPS samples, significantly so in the MPS cohort as a whole (p = 0.04; control 1.4μg ml^-1^ vs MPS 8.9μg ml^-1^) and contributed to predominantly by MPS VI—demonstrating a 12-fold increase over control samples (p = 0.0007).

#### Lysosomal compartment size in MPS patient samples

Lysosomal storage was demonstrated and quantified with LAMP2 and compared to control in adenotonsillar tissue of patients (Fig 4). LAMP2 reactivity demonstrated a vesicular pattern of staining. In patients, lysosomal compartment size was significantly increased in the MPS cohort as a whole (t-test, p = 0.0007; Fig 5) and in all individual MPS types compared to control (ANOVA; MPS I, p = 0.006; III, p = 0.0007; IV p = 0.004; VI, p = 0.04). Staining was especially prominent in the mantle and interfollicular zones and less so in the germinal centre as demonstrated in the representative sections. However, the exception to this was the untreated MPS III patient, who was noted to have excess lysosomal storage within the germinal centre of the lymphoid follicles, suggesting additional involvement of the leukocyte population. In this individual, LAMP2 was seen to co-localise with HS within the follicular stroma in addition to the endothelial walls as seen elsewhere. The untreated MPS IIIA patient was noted to have significantly increased lysosomal storage in comparison to the other ERT treated MPS types (MPS III vs. MPS I, p = 0.04; IV, p = 0.03; VI, p = 0.04). A significant correlation between LAMP2 and HS intensity was identified in individuals confirming the perceived pathological association between primary GAG accumulation and lysosomal storage burden (R^2^ = 0.44, p = 0.01; Fig 5).

### Quantitative RT-PCR of pro-inflammatory cytokines in patients

**Fig 6:**
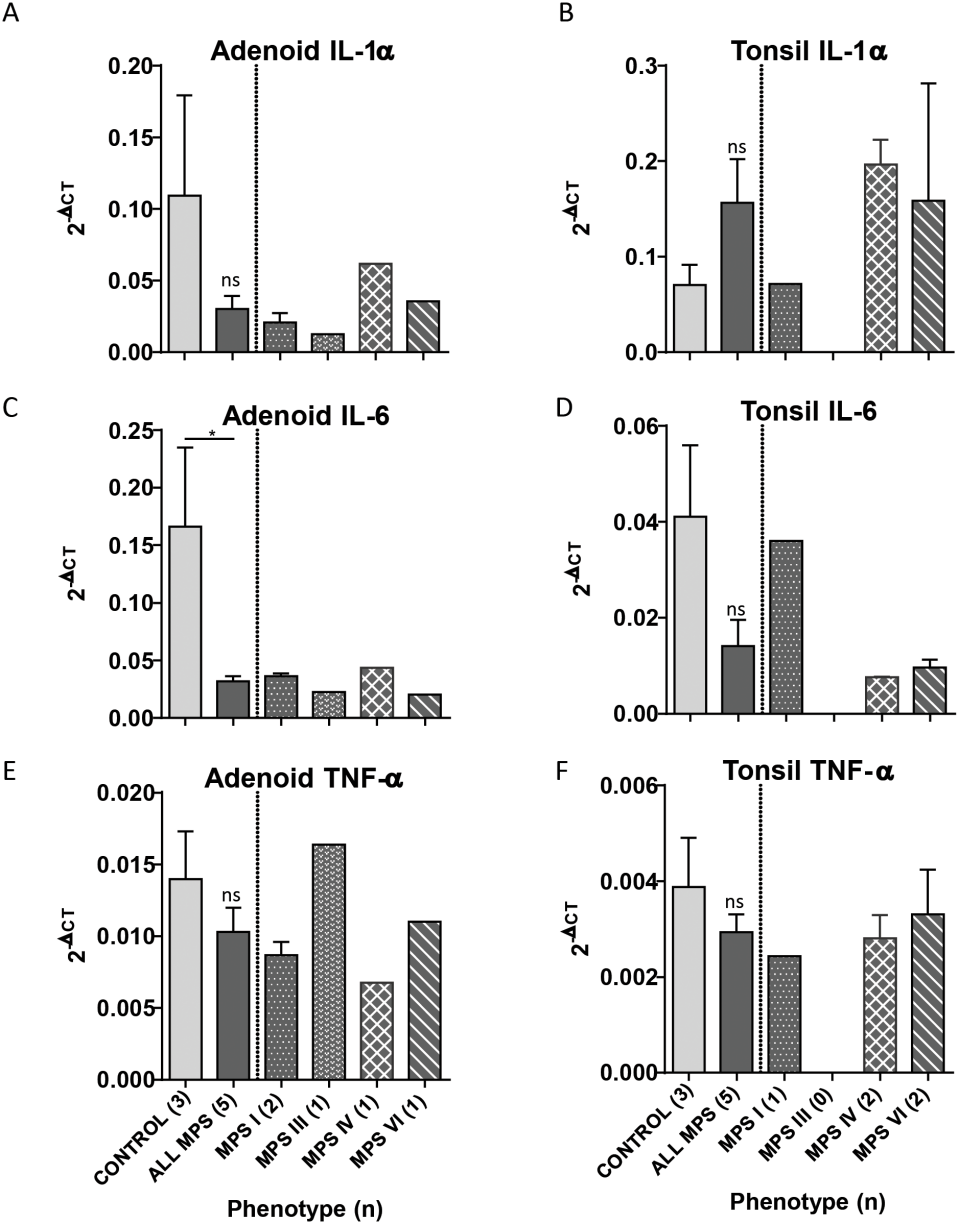
Reduced cytokine expression profiles in adenotonsillar tissue of MPS patients. Cytokine profile was determined by real time RT-PCR. RNA from adenoid (A, C, E) and tonsil (B, D, F) were extracted and quantified for (A-B) IL-1α, (C-D) IL-6, (E-F) TNF-α (n for each patient group in parentheses) using the comparative C_T_ method and normalised to GAPDH. Adenoid and tonsil tissue were analysed and presented separately as tonsil was shown to have persistently raised expression compared to adenoid in the same patient. The 2^−ΔCT^ values are presented for comparison. IL-6 was significantly reduced in MPS in comparison to control tissue from unaffected individuals. Means are presented with error bars representing SEM. Statistical analysis was performed using student’s t-test for the group as a whole and one-way ANOVA with Tukey’s multiple comparisons test for individual types. Between groups demonstrated with solid bars * p<0.05, **p<0.01, ***p<0.001, ns = not significant for total MPS cohort.

To determine the relative gene expression of pro-inflammatory cytokines in MPS compared with unaffected controls, quantitative RT-PCR using the comparative C_T_ method was used. As the control samples analysed were not paired to MPS samples, relative expression was conveyed as the mean 2^-ΔCT^ and variability between MPS and control groups (Fig 6). Table 2 presents the fold change in expression in relation to the control sample cohort.

**Table 2:** Relative gene expression in MPS tissue; expressed as fold difference.

	All MPS	MPS I	MPS III	MPS IV	MPS VI
***Adenoid***					
**IL-1α**	-3.60	-5.25	-8.69	-1.77	-3.07
**IL-6**	-5.23*	-4.59	-7.35	-3.83	-8.12
**TNF-α**	-1.36	-1.61	1.17	-2.06	-1.27
***Tonsil***					
**IL-α**	2.22	1.02		2.79	2.26
**IL-6**	-2.91	-1.14		-5.38	-4.27
**TNF-α**	-1.32	-1.59		-1.38	-1.17

*p<0.05.

Comparison of expression between tonsil and adenoid tissue taken from the same individual (n = 5) demonstrated consistently raised expression of cytokines in tonsils relative to adenoids, although this failed to reach significance (tonsils relative to adenoids; IL-1α: 3.6 fold; IL-6: 1.2 fold; TNF-α: 1.3 fold). As a result, adenoid and tonsil data are presented separately. A significant degree of variability in expression in our control samples was seen, raising the possibility of subclinical infection in some patients.

Analysis demonstrated a reduction in expression of our pro-inflammatory cytokine panel in relation to control samples for all cytokines in adenoid tissue and IL-6 and TNF-α in tonsil. This reduction was significant for IL-6 in adenoid tissue (5.2 fold reduction, p = 0.038).

## Discussion

Our study performs the most detailed assessment to date of the structural and biochemical pathobiology within tissues contributing to upper airway obstruction in MPS. We find evidence of persistent lysosomal and GAG storage in adenotonsillar tissue despite ongoing treatment with ERT. We further observe significant alteration in ECM deposition, suggesting tissue remodelling occurring as a secondary process responsible for disease pathogenesis. We were unable to identify evidence of increased inflammation within these tissues.

The characteristic architecture of adenotonsillar tissue, composed of a reticular fibre meshwork of various ECM proteins in close proximity to follicular lymphatic structures appears to be preserved in MPS. Previous studies of adenotonsillar tissue in MPS have focused solely on these histological appearances or crude GAG quantification [35–37]. We have confirmed the primary pathological defect of lysosomal storage and GAG accumulation within the airway. Lysosomal storage in patients appears to localise most prominently to the subepithelial regions described as containing aggregations of histiocytoid cell forms, identified as pathognomonic of MPS on adenotonsillar histology [36]. Significantly, the lysosomal compartment was persistently enlarged in patients, despite treatment with ERT. Despite being a well vascularized tissue—and so well supplied with intravenous recombinant enzyme—our observations confirm the fact that current treatment with ERT fails to fully reverse adenotonsillar storage pathology in MPS, manifesting with ongoing clinical disease. Of note, the untreated patient with MPS IIIA was found to have higher intensity LAMP2 staining compared to ERT treated individuals, suggesting a partial response following treatment in other MPS types.

Collagen IV and laminin α-5 are the most widely expressed ECM components in the follicles, interfollicular zone and capillary network of lymphoid tissue [29] and our observations confirm they are widely expressed. However, despite preserved follicular architecture on histology, we demonstrate increased staining of both collagen IV and laminin α-5 in adenotonsillar samples from MPS patients in comparison to controls. A denser pattern of staining within the reticular and capillary basement membrane network appears responsible for this difference. These observations suggest ECM remodelling and possibly altered angiogenesis in adenotonsillar tissue in MPS. We hypothesise these changes to be responsible for not only the increased incidence of hypertrophy causing obstruction, but also the high regrowth rate in adenoid tissue commonly necessitating revision surgery [15].

As has previously been determined in the CNS of MPS mice models [31], secondary storage events contribute to disease pathogenesis. Within the airway, altered matrix behaviour is likely to be explained via the role of GAGs and their proteoglycans (PGs) in potentiating interactions with ECM proteins and growth factors—such as fibroblast and vascular endothelial—responsible for angiogenesis and ECM deposition [38, 39]. Their critical role within the airway has been demonstrated in asthmatics, with collagen and PG deposition contributing to bronchial remodelling and hyper-responsiveness [40, 41]. The relationship between HS PGs and collagen integrity is well demonstrated in musculoskeletal disease [42, 43]. The presence of ECM dysregulation in early stage MPS disease has recently been identified in a study of chondro-osseus disease in MPS I mice [44] and identified in case reports from corneal analysis in MPS I, IV and VII [45, 46]. Presumably, failure to degrade GAGs and their PGs through normal lysosomal pathways in MPS results in secretion, deposition and interaction in extracellular matrix.

Examination of serial sections demonstrates that pathological storage of HS appears to co-localise with collagen and laminin within the BM and reticular network of the tonsils and adenoids, thus making direct interaction more likely. This is in keeping with findings that PGs localise to the BM in tonsil [47] while studies have demonstrated that induction of PG synthesis is reliant on a surrounding collagen matrix [48]. While the role of GAGs and matrix metalloproteinases on remodelling in MPS has been investigated in animal models of joint disease, such an interaction potentially explains the ongoing burden of disease seen in patients while the mechanism merits further investigation within the airway [49].

Clinical and observational studies in the literature, including a systematic review of pre and post ERT polysomnography measures of obstruction, fail to demonstrate a significant improvement in OSA severity following commencement of ERT [23, 50, 51], The clinical implication of our findings suggests that early diagnosis and commencement of ERT, prior to the development of adenotonsillar hypertrophy and significant obstruction, may prevent progression to significant SDB. However, once obstructive adenotonsillar hypertrophy and SDB are present, ERT is unlikely to reverse or normalise this, and thus early surgical intervention or ventilatory support in the management upper airways obstructive disease would be indicated.

Currently, there is no existing clinical or experimental evidence of altered immune function in the airway of patients with MPS. The interactions between pro and anti-inflammatory cytokines and GAGs and their PGs is complex [52]. The cytokines assessed were specifically chosen as they have been shown to be involved in inflammatory pathways in MPS, while also being critical to respiratory host defence to infection [31, 53, 54]. They represent acute phase macrophage mediated inflammation implicated in studies investigating the neuropathology of MPS disease. These indicate an upregulation in inflammatory cytokines in the peripheral circulation and in brains of untreated MPS I, IIIA and IIIB mice. Specific pathological modifications to GAG, including alterations to sulphation patterns [27] and secondary storage are thought to elicit this inflammation [21, 52]. Adenotonsillar lymphoid tissue of Waldeyer’s ring forms part of the mucosa-associated lymphoid system, exposed to airborne and alimentary antigens. The predominantly active population within the germinal centres are antigen stimulated B-cells, which subsequently undergo differentiation and clonal expansion to immunoglobulin (IgG and IgA) producing plasma cells responsible for secretory immunity as part of an adaptive response [55]. Relatively little is known about perturbations in adaptive immunity in MPS [22].

Quantitative PCR was unable to show a significant upregulation in pro-inflammatory cytokine expression in MPS airway samples. In fact, relative expression of cytokines responsible for innate immunity was generally reduced in adenotonsillar tissue in comparison to control samples. This was exaggerated by a significant degree of variability amongst the control patient cohort, despite a history of tonsillitis being sought as an exclusion criterion. IL-6 plays both pro and anti-inflammatory roles, including in the acute response to microbial infection and is required for the transition from innate to acquired immunity [56]. Interestingly, studies have previously shown that IL-6 inhibits PG synthesis in vitro chondrocytes [57]. Hence, it is possible that the reduced cytokine expression profile found in our samples facilitates excess PG and ECM deposition seen in MPS patients. Given the lack of an overt primary pro-inflammatory response, the features of reactivity seen clinically, likely occur secondary to obstruction with impaired mucociliary clearance and retained secretions acting as a stimulus towards microbial colonisation and inflammation rather than an innate inflammatory upregulation [58–61].

Analysis of patient samples alone has a number of drawbacks. The primary limitation of clinical studies of a rare and heterogeneous disease, such as MPS, is the small population available for recruitment. Infrequent surgery given the anaesthetic risks means that tissue availability is significantly limited. Indeed, no patients with MPS II underwent adenotonsillar surgery during the study recruitment period in our institution. Additionally, all individuals except MPS III and IVB patients are currently treated with either ERT or HSCT and hence patient samples were unavailable to provide comparative information on pre-therapeutic manifestations of disease. Determination of the untreated and lower respiratory tract phenotype maybe facilitated by use of a murine model of MPS, however it remains unclear whether mouse models of MPS provide an accurate reflection of airway manifestations seen in patients.

## Conclusion

We have performed a detailed analysis of airway pathobiology in patient samples, identifying novel findings that potentially explain the phenotype of airway disease in MPS. While ERT is known to reverse features such as hepatosplenomegaly, previous clinical and observational studies [23, 50, 51] have shown that once established, upper airway obstruction and OSA do not significantly improve or resolve following commencement of ERT. To explain this phenomenon, we identify persistently increased primary storage pathology in patient tissue despite treatment with ERT. We additionally show features consistent with ECM remodelling in adenotonsillar tissue, that appear persistent and irreversible despite ERT, and contribute to persistent adenotonsillar hypertrophy and airway obstruction. Finally, we find no evidence of primary upregulation in inflammation at homeostasis, suggesting manifestations of inflammatory disease noted within the respiratory tract occur secondary to structural alterations and impaired function.

As a consequence of these finding, we recommend that efforts be directed towards earlier diagnosis and initiation of ERT, for example with neonatal metabolic screening, to prevent the early and progressive substrate accumulation and that once established leads to irreversible and persistent upper airways obstruction. Secondly, once established and if significant, interventions, such as surgical adenotonsillectomy or non-invasive ventilatory support, in addition to ongoing disease modifying therapy, be targeted at relieving the obstructive manifestations noted within the airway of MPS.

## Supporting information

S1 Table Collagen IV immunoreactivity quantified in adenotonsillar samples from 8 MPS patients and compared to samples from 4 non-affected control individuals using Image J to calculate mean staining intensity from representative sections. (XLSX)

S2 Table Laminin α-5immunoreactivity quantified in adenotonsillar samples from 8 MPS patients and compared to samples from 4 non-affected control individuals using Image J to calculate mean staining intensity from representative sections. (XLSX)

S3 Table LAMP 2immunoreactivity quantified in adenotonsillar samples from 8 MPS patients and compared to samples from 4 non-affected control individuals using Image J to calculate mean staining intensity from representative sections. (XLSX)

S4 Table Heparan sulphate immunoreactivity quantified in adenotonsillar samples from 8 MPS patients and compared to samples from 4 non-affected control individuals using Image J to calculate mean staining intensity from representative sections. (XLSX)

S5 Table Total GAG determined using the Blyscan assay. (XLSX)

S6 Table Relative expression of inflammatory cytokines, IL-1α, IL-6, and TNF-α using quantitative PCR—ΔCт, ΔΔCт and 2^−ΔCT^ are provided. (XLSX)

S7 Table Relative expression of inflammatory cytokines, IL-1α, IL-6, and TNF-α determined from raw data provided in S6 Table. (XLSX)
